# Supplementary material for: Predicting visual function by interpreting a neuronal wiring diagram
Source: Nature. 2024 Oct 2;634(8032):113–23. doi: 10.1038/s41586-024-07953-5 (PMC11446822; doi:10.1038/s41586-024-07953-5)
Supplement: Supplementary file 6 — CRF and ERF predictions for individual TmY4 and TmY9 cells. Analogous to Supplementary Data 3, but for TmY target types. Shown are the top four monosynaptic pathways, the strongest pathway passing through each of the top ten intermediary types (ranking from Extended Data Fig. 7), and the trisynaptic pathway Tm1–TmY–Dm3–TmY (see the section entitled Prediction of spatial normalization). [file 41586_2024_7953_MOESM6_ESM.zip › DataS4/TmY9qΓèÑ/720575940614117039.pdf]

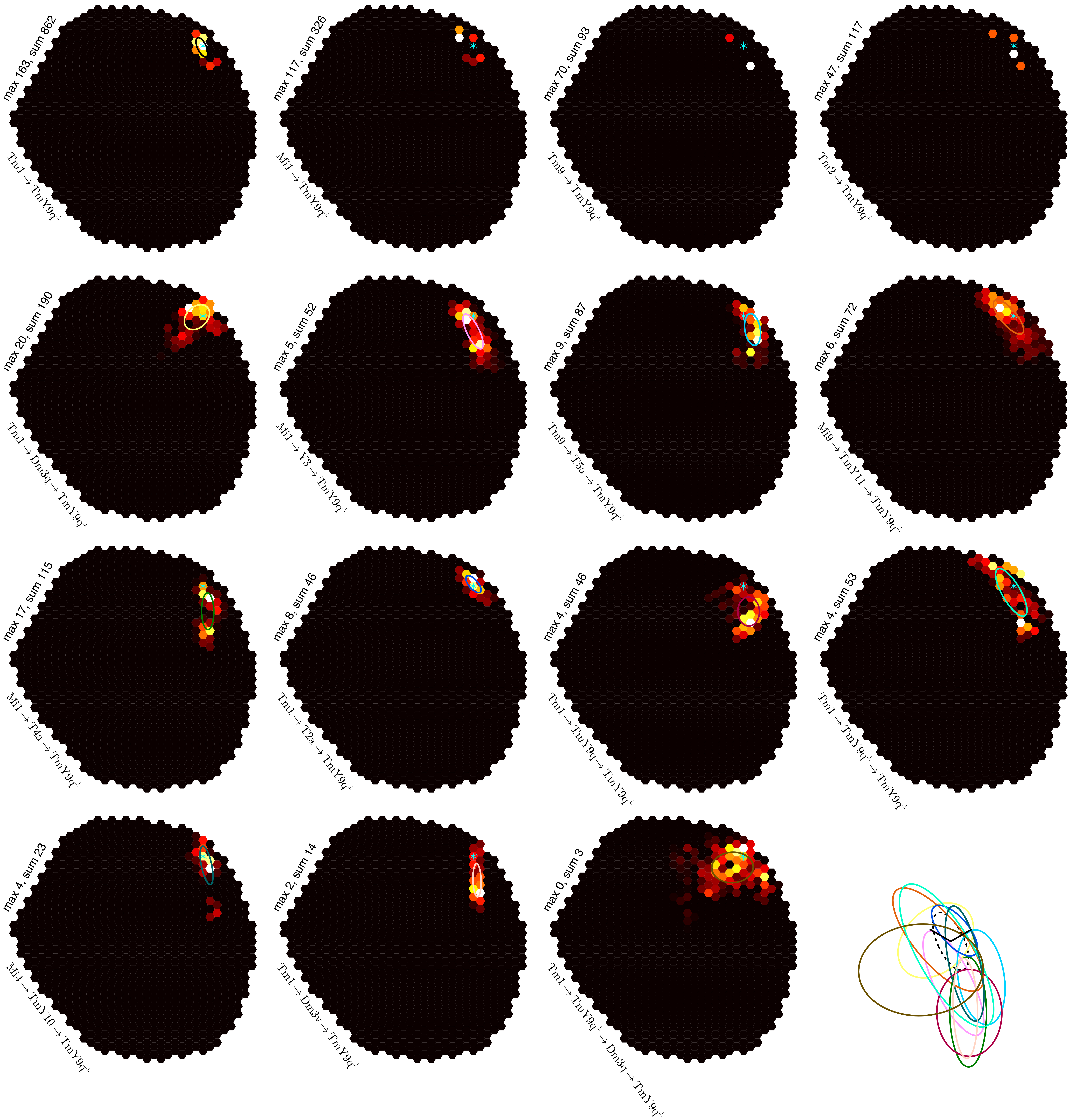
max 163, sum 862
Tm1 → TmY9q⊥
max 117, sum 326
Mi1 → TmY9q⊥
max 70, sum 93
Tm9 → TmY9q⊥
max 47, sum 117
Tm2 → TmY9q⊥
max 20, sum 190
Tm1 → Dm3q → TmY9q⊥
max 5, sum 52
Mi1 → Y3 → TmY9q⊥
max 9, sum 87
Tm9 → T5a → TmY9q⊥
max 6, sum 72
Mi9 → TmY11 → TmY9q⊥
max 17, sum 115
Mi1 → T4a → TmY9q⊥
max 8, sum 46
Tm1 → T2a → TmY9q⊥
max 4, sum 46
Tm1 → TmY9q → TmY9q⊥
max 4, sum 53
Tm1 → TmY9q⊥ → TmY9q⊥
max 4, sum 23
Mi4 → TmY10 → TmY9q⊥
max 2, sum 14
Tm1 → Dm3v → TmY9q⊥
max 0, sum 3
Tm1 → TmY9q⊥ → Dm3q → TmY9q⊥
